# Induction of the Coxsackievirus and Adenovirus Receptor in Macrophages During the Formation of Atherosclerotic Plaques

**DOI:** 10.1093/infdis/jiaa418

**Published:** 2020-07-10

**Authors:** Azadeh Nilchian, Estelle Plant, Malgorzata M Parniewska, Ana Santiago, Aránzazu Rossignoli, Josefin Skogsberg, Ulf Hedin, Ljubica Matic, Jonas Fuxe

**Affiliations:** 1 Department of Laboratory Medicine, Division of Pathology, Karolinska Institutet and Karolinska University Hospital Huddinge, Stockholm, Sweden; 2 Department of Microbiology, Tumor and Cell Biology, Karolinska Institutet, Stockholm, Sweden; 3 Department of Medical Biochemistry and Biophysics, Karolinska Institutet, Stockholm, Sweden; 4 Department of Molecular Medicine and Surgery, Karolinska Institutet and Karolinska University Hospital Solna, Stockholm, Sweden

**Keywords:** atherosclerosis, enterovirus, coxsackievirus, virus receptors, macrophages, CXADR/CAR

## Abstract

Multiple viruses are implicated in atherosclerosis, but the mechanisms by which they infect cells and contribute to plaque formation in arterial walls are not well understood. Based on reports showing the presence of enterovirus in atherosclerotic plaques we hypothesized that the coxsackievirus and adenovirus receptor (CXADR/CAR), although absent in normal arteries, could be induced during plaque formation. Large-scale microarray and mass spectrometric analyses revealed significant up-regulation of CXADR messenger RNA and protein levels in plaque-invested carotid arteries compared with control arteries. Macrophages were identified as a previously unknown cellular source of CXADR in human plaques and plaques from *Ldr*^*−/−*^*Apob*^*100/100*^ mice. CXADR was specifically associated with M1-polarized macrophages and foam cells and was experimentally induced during macrophage differentiation. Furthermore, it was significantly correlated with receptors for other viruses linked to atherosclerosis. The results show that CXADR is induced in macrophages during plaque formation, suggesting a mechanism by which enterovirus infect cells in atherosclerotic plaques.

## Teaser

Virus infections are linked to atherosclerosis but the mechanisms are unclear. We found that the coxsackievirus and adenovirus receptor is induced in macrophages during plaque formation in arterial walls, suggesting a mechanism for how enterovirus infect cells in atherosclerotic plaques.

Atherosclerosis is a chronic inflammatory disease involving macrophage infiltration into arterial walls where they take up oxidized low-density lipoprotein and form foam cells and plaques. What drives the chronic inflammation that leads to the development of plaques is still not clear [1]. An emerging paradigm suggests that pathogens including viruses and bacteria contribute to the pathogenesis of atherosclerosis [2]. As highlighted recently, virus infections are also associated with plaque disruption and myocardial infarction [3].

A role for viruses in atherosclerosis was suggested in the 1970s when it was discovered that herpesvirus infection promotes atherosclerosis in chickens [4]. Subsequent studies have shown that various viruses can promote atherosclerosis in animal models [5]. A possible explanation is that viruses promote plaque formation indirectly by for example triggering systemic release of inflammatory cytokines and acute phase proteins. However, a number of viral pathogens, including enterovirus, cytomegalovirus, hepatitis C virus, human immunodeficiency virus (HIV), and herpes simplex virus, have actually been detected locally in atherosclerotic plaques [5], suggesting that these viruses may directly target cells within the plaques. Furthermore, recent data also imply that coinfections with multiple viruses, that is, the total “infectious burden," is a higher risk factor for atherosclerosis than the presence of one infectious agent [6].

Enterovirus RNA has been isolated from atherosclerotic lesions [7] and is associated with an increased risk of developing unstable angina [8], and acute myocardial infarction [9]. In one study, enterovirus antigens were detected in 49% of patients with coronary heart disease and in 54.3% of those with myocardial infarction [10]. Experimental studies have shown that coxsackievirus B (CVB), a subspecies of the enterovirus family, can promote atherosclerosis in animal models [11], yet the mechanisms of how CVB and other enteroviruses target the plaque microenvironment are not clear.

The coxsackievirus and adenovirus receptor (CXADR/CAR) is the high-affinity receptor for CVB and other subtypes of enteroviruses [12]. CXADR is not expressed in normal blood vessels, and its expression in atherosclerotic vessels has not been studied. CXADR belongs to the cortical thymocyte marker in the *Xenopus* (CTX) family of tight junction–associated transmembrane proteins that are involved in cell adhesion [13, 14]. Among CTX proteins, CXADR is unique, because it is essential for normal development—CXADR-deficient mouse embryos die of heart failure [15]. CXADR has been identified as a key regulator of the AKT signaling pathway [16] and of epithelial-mesenchymal transition in breast cancer cells [17]. Considering the fact that CXADR is deregulated in various cancer and inflammatory conditions [18], we hypothesized that CXADR expression might be induced during plaque formation in arteries.

## MATERIALS AND METHODS

### Human Atherosclerotic Plaques

Human endarterectomy samples were collected from patients undergoing surgery for asymptomatic or symptomatic high-grade (>50% The North American Symptomatic Carotid Endarterectomy Trial [NASCET] [19]) carotid stenosis, and are part of the Biobank of Karolinska Endarterectomies. Symptoms of plaque instability were defined as transient ischemic attack, minor stroke, and amaurosis fugax. Samples were analyzed by means of gene microarrays (n = 127) or mass spectrometry (n = 18). Control arteries (iliac and radial arteries and 1 aorta) were obtained from organ donors without any history of cardiovascular disease. All samples were collected with informed consent from patients or organ donors’ guardians according to the Declaration of Helsinki and with the approval of the Ethical Committee of Northern Stockholm. The methods used during the study of human samples were carried out in accordance with the ethical guidelines and regulations.

### Gene Expression Analysis of Human Plaques

For microarrays, plaques (n = 127) were divided transversally at the most stenotic part, with the proximal half of the lesion used for RNA preparation, as described elsewhere [20], while the distal half was fixed in 4% zinc formaldehyde and processed for histology. RNA was extracted using Qiazol Lysis Reagent (no. 79306; Qiagen) and purified with the RNeasy Mini kit (no. 74106; Qiagen). The concentration was measured using a NanoDrop ND-1000 spectrophotometer (Thermo Scientific) and the quality estimated by a Bioanalyzer capillary electrophoresis system (Agilent Technologies). Total RNA of appropriate quality, purity, and integrity (RNA integrity number, 7–10; absorbance at 260/280 nm, 1.7–2.0, absorbance at 260/230 nm, 0.7–1.5) was used for microarray transcript profiling with Affymetrix arrays. The microarray data set is available from Gene Expression Omnibus (GSE21545).

### Proteomic Analysis of Human Plaques

For proteomic analyses performed using liquid chromatography with tandem mass spectrometry, atherosclerotic plaques from 18 patients (9 symptomatic and 9 asymptomatic; matched for sex, age, and statin medication) were processed as described elsewhere [21]. A central portion of the plaque corresponding to the maximum stenosis was separated from the respective downstream peripheral end (adjacent tissue) of the plaque and used in comparisons.

### Immunohistochemistry

Immunohistochemistry on human tissues was performed as described elsewhere [22]. All reagents were from Biocare Medical. Isotype rabbit and mouse immunoglobulin G serum were used as negative controls. In brief, 5-μm sections were deparaffinized in Tissue Clear (Sakura, Gothenburg, Sweden) and rehydrated in ethanol. For antigen retrieval, slides were subjected to high-pressure boiling in DIVA buffer (pH 6.0). After blocking with Background Sniper reagent, primary antibodies were diluted in Da Vinci Green solution, applied on slides, and incubated at room temperature for 1 hour. For costaining, a double-stain probe-polymer system containing alkaline phosphatase and horseradish peroxidase was applied, with subsequent detection using Warp Red and Vina Green stains. Slides were counterstained with Hematoxylin QS (Vector Laboratories), dehydrated, and mounted in Pertex (Histolab). Images were taken with a Nikon OPTIPHOT*-*2 microscope equipped with a digital camera and processed with NIS-Elements software (Ver 3.22.14).

### Differentiation and Polarization of THP-1 Monocytes

THP-1 cells (American Type Culture Collection) were cultured at 37°C with 5% carbon dioxide in Roswell Park Memorial Institute 1640 medium (no. 2187509; Thermo Fisher Scientific) containing 10% fetal bovine serum. To differentiate monocytes into macrophages (M0), cells were incubated for 24 hours with 150 nmol/L phorbol 12-myristate 13-acetate (no. P8139PMA; Sigma/Merck), followed by 24 hours in Roswell Park Memorial Institute 1640 medium. M0 macrophages were consequently incubated with 20 ng/mL inteferon γ (no. PHC4031; Thermo Fisher Scientific) and 10 pg/mL lipopolysaccharide (no. 2630; Merck) for 24 hours, or with 20 ng/mL interleukin 4 (no. 200–04; Peprotech) and 20 ng/mL interleukin 13 (no. 213-ILB; R&D Systems) for 72 hours, for polarization into M1 or M2 macrophages, respectively.

### Western Blot Analysis

Cells were lysed in RIPA buffer (no. 89900; Thermo Fisher Scientific) supplemented with protease and phosphotase inhibitors (no. 87785; Thermo Fisher Scientific) and total protein extracts were boiled in Laemmli sample buffer (no. NP0007; Thermo Fisher Scientific), separated by sodium dodecyl sulfate–polyacrylamide gel electrophoresis under reducing conditions and transferred to nitrocellulose using the iBlot2 system (Thermo Fisher Scientific). The membrane was blocked using blocking reagent (no. 11520709001; Roche Diagnostics) for 1 hour at room temperature and then incubated with a primary antibody against CXADR (no. AF3336; goat anti-hCAR; R&D Systems) overnight at 4°C. Subsequently, the membrane was incubated with a horseradish peroxidase–conjugated donkey anti-goat anti–immunoglobulin G secondary antibody (no. PA1-28664; Thermo Fisher Scientific) for 1 hour at room temperature. Immunoreactive bands were visualized by means of chemiluminescence (ChemiDoc XRS+; Image Lab Software; Bio-Rad). Blots were checked for equal loading by probing with a rabbit polyclonal anti-calnexin antibody [14].

### Immunofluorescence Staining

Hearts were isolated from *Ldlr*^−/−^*Apob*^100/100^*Mx1CreMttp*^flox/flox^ mice at 10 (n = 3) and 50 (n = 3) weeks of age that were perfused with 2% paraformaldehyde in phosphate-buffered saline (PBS) at the time the mice were euthanized. Tissues were frozen in OCT compound (no. 00890; Histolab) and cut in 5-µm aortic root cryosections. Sections were fixed in acetone, blocked in 10% donkey serum plus 4% bovine serum albumin (nos. D9663 and A2153; Merck) in PBS, costained with primary antibodies against CXADR (no. AF2654; R&D Systems), CD68 (no. MCA1957; Bio-Rad), and CD31 (no. 32457; Abcam) at 4°C overnight and Alexa 488, 594, and 647 secondary antibodies (Thermo Fisher Scientific) for 3 hours at room temperature, washed, and mounted with Vectashield DAPI (no. H-1800; Vector Laboratories).

THP-1 cells (4 × 10^5^) were seeded in 24-well plates with coverslips and subjected to differentiation and macrophage polarization as described above. Cells were then fixed with 95% ice-cold ethanol for 30 seconds, blocked in 5% donkey serum plus 2% bovine serum albumin for 1 hour, and stained overnight with a rabbit anti-CXADR (SIV) antibody [14] diluted 1:10 in PBS, followed by an Alexa Fluor 488 goat anti-rabbit secondary antibody (no. A32731; Thermo Fisher Scientific) diluted 1:1000 in PBS for 90 minutes. Images were captured by a Zeiss LSM700 confocal microscope.

### Mouse Model

Transgenic *Ldlr*^−/−^*Apob*^100/100^*Mx1Cre Mttp*^flox/flox^ mice were used without activating the Cre-lox system and are referred to as *Ldlr*^−/−^*Apob*^100/100^ mice. Male mice were housed in a pathogen-free barrier facility, where they were fed rodent chow containing 4% fat to promote atherosclerosis, as described elsewhere [23]. Mouse protocols were approved by the Ethical Committee of Northern Stockholm and were carried out in accordance with the ethical guidelines and regulations.

### Statistical Analysis

Transcriptomic and proteomic data set analyses were performed with GraphPad Prism 6 and Bioconductor software using a linear regression model adjusted for age and sex or a 2-sided Student *t* test assuming nonequal deviation, with correction for multiple comparisons according to Bonferroni. Pearson or Spearman correlations were calculated to determine the association between messenger RNA (mRNA) and protein expression levels from microarrays and liquid chromatography with tandem mass spectrometry. Correlation coefficient (*r*) values were considered strong (±0.6–1.0), moderate (±0.4–0.6), or weak (±0.2–0.4). Differences were considered statistically significant at *P* < .05.

## RESULTS

### Up-regulation of CXADR in Human Carotid Plaques

**Figure 1.**
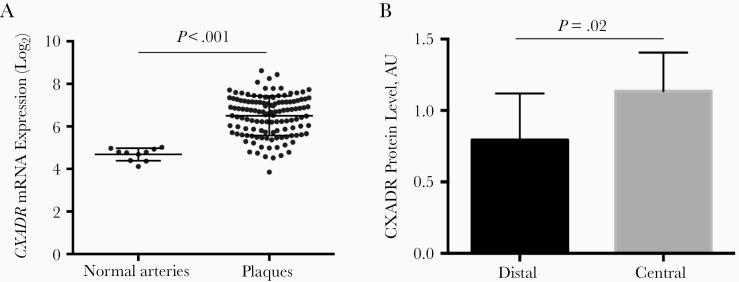
Up-regulation of coxsackievirus and adenovirus receptor (CXADR) in human carotid plaques. *A,* Plot graph showing *CXADR* messenger RNA (mRNA) expression in human carotid plaques (n = 127) compared with normal arteries (n = 10). *B,* Bar graph showing results from proteomic analysis of CXADR protein levels in plaques (central) versus nonplaque regions (distal) of human carotid arteries. Abbreviation: AU, arbitrary units.

Large-scale microarray analysis was performed to study the expression of *CXADR* mRNA in atherosclerotic plaques from patients with carotid stenosis (n = 127). The expression of *CXADR* was significantly increased in carotid arteries with atherosclerotic plaques (*P* < .001; mean log_2_ difference [standard error of the mean], 1.82 [0.12]) compared with normal arteries (Figure 1A). This finding was supported by proteomic data showing increased CXADR protein levels in plaques compared with adjacent, control arterial tissue (*P* = .02; mean difference [standard error of the mean], 0.34 [0.12]) (Figure 1B). No significant difference in CXADR expression in carotid arteries could be detected between patients with asymptomatic and those with symptomatic disease (Supplementary Figure 1).

### Induction of CXADR During Plaque Formation in *Ldr*^*−/−*^*Apob*^*100/100*^ Mice

**Figure 2.**
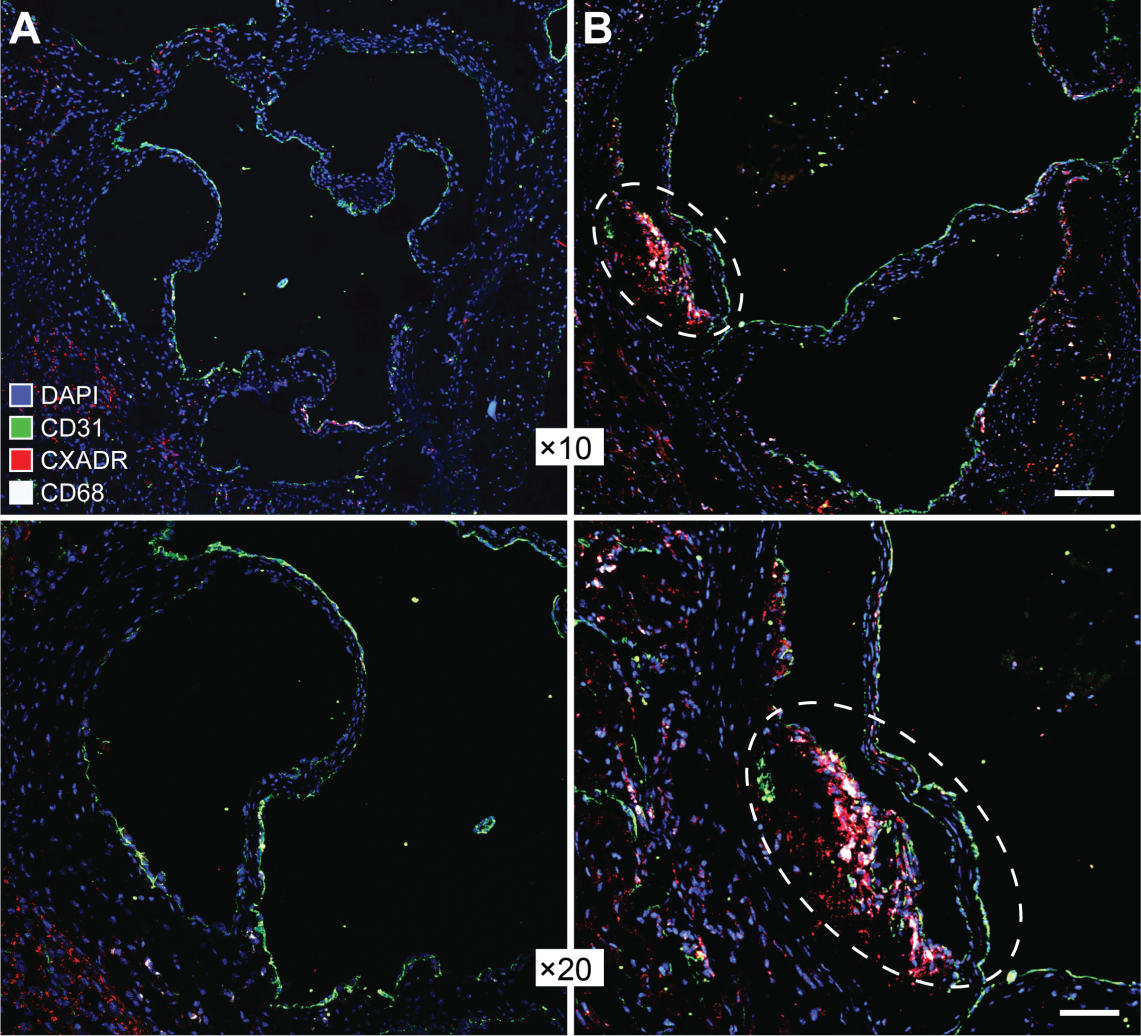
Up-regulation of coxsackievirus and adenovirus receptor (CXADR) in aortic plaques in a mouse model of atherosclerosis. Representative immunofluorescent images showing staining of CXADR (*red*) in aortic walls from 10-week-old (*A*) or 50-week-old (*B*) *Ldr*^*−/−*^*Apob*^*100/100*^ mice. Endothelial cells and macrophages were stained with antibodies against CD31 (*green*) and CD68 (*white*), respectively. Cell nuclei were labeled with DAPI (*blue*). A plaque lesion in the aortic wall of a 50-week-old mouse is enclosed in a dashed oval (*right-hand images*). Images were obtained with a digital camera and processed with NIS-Elements software (version 4.0; https://www.microscope.healthcare.nikon.com/products/software/nis-elements). Scale bars represent 60 μm (×10 magnification images) and 30 μm (×20 magnification images).

To obtain a more longitudinal view on CXADR expression during plaque formation we used immunostaining to analyze aortic walls from transgenic *Ldr*^*−/−*^*Apob*^*100/100*^ mice of different ages. In this animal model of atherosclerosis, plaque formation is first seen in the aortic root at about 20 weeks and progresses up to 60 weeks [24]. In line with this, we observed plaques in aortas from 50-week-old *Ldr*^*−/−*^*Apob*^*100/100*^ mice, but not in 10-week-old mice (Figure 2). Similar to the results obtained from analyzing human plaques, we observed CXADR staining in plaque-invested aortic walls of 50-week old mice but not in 10-week old mice. Moreover, we found that CXADR staining in plaques colocalized with the macrophage marker CD68, suggesting that macrophages could represent a cellular source of CXADR in atherosclerotic plaques.

### Macrophages as a Cellular Source of CXADR in Atherosclerotic Plaques

**Figure 3.**
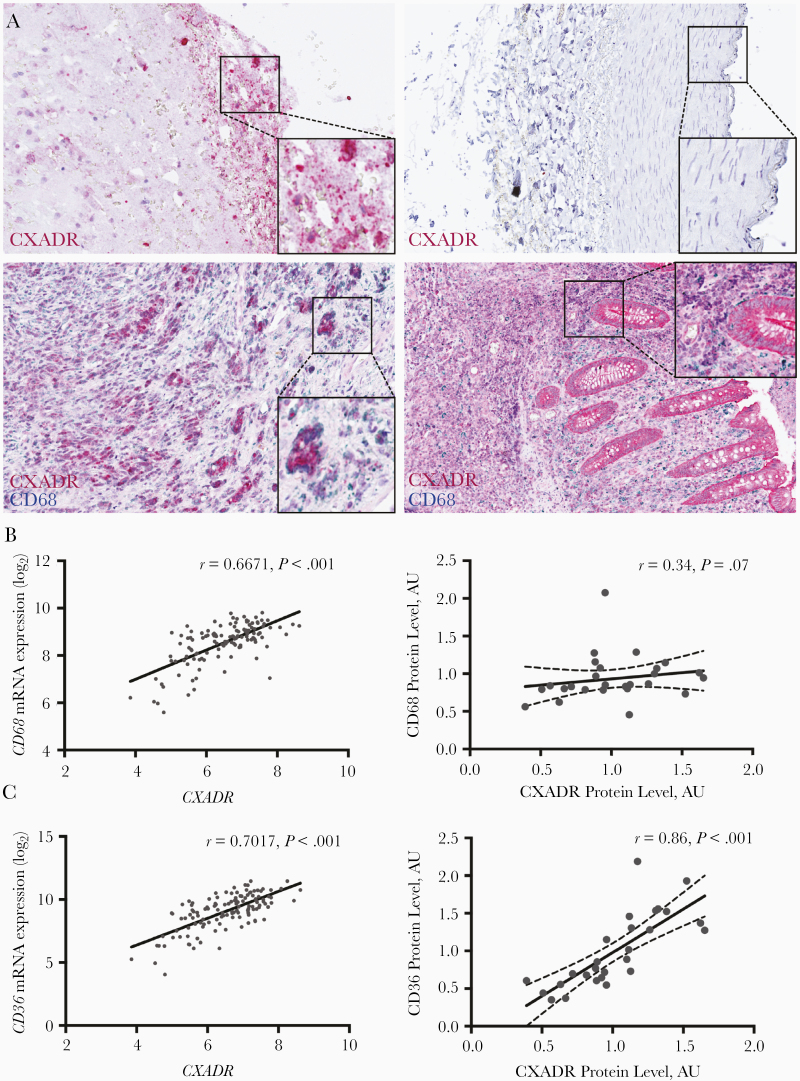
Immunohistochemical analysis of coxsackievirus and adenovirus receptor (CXADR) expression and localization in human carotid plaques. *A,* Sections of a carotid artery containing a plaque (*upper and lower left*), a normal artery (*upper right*), and the appendix (*lower right*) were stained with antibodies against CXADR (*upper panels*), or the combination of CXADR and CD68 (*lower panels*). *B, C,* Pearson correlation analysis showing the association between CXADR and CD68 (*B*) and between CXADR and CD36 (*C*) in human carotid plaques at messenger RNA (*left*) and protein (*right*) levels. Abbreviation: AU, arbitrary units.

To further analyze the localization of CXADR in human plaques we performed immunohistochemical staining. Positive staining was detected in carotid plaques, but not in normal arteries (Figure 3A). Costaining revealed, similar to what was observed in the mouse model, that CXADR-positive cells were positive for CD68. However, not all CD68-positive cells stained positive for CXADR, suggesting that CXADR expression in plaques was restricted to certain subpopulations of macrophages. For comparison, we costained human appendix for CXADR and CD68. As shown elsewhere [14], CXADR was detected in intestinal epithelial cells. In contrast, CD68-positive cells in the appendix did not stain positive for CXADR. Further analysis of carotid plaques revealed that CXADR and CD68 were correlated at mRNA (*r* = 0.6671; *P* < .001), but not at protein levels (*r* = 0.34; *P* < .07) (Figure 3B). Together, these results suggested that CXADR is not expressed in all CD68-positive cells but is expressed more specifically in subpopulations of CD68-positive cells in atherosclerotic plaques with increased *CD68* mRNA production.

Different subpopulations of macrophages exist in atherosclerotic plaques and are often classified as being proinflammatory (M1), anti-inflammatory (M2), or variants thereof [25, 26]. M1 macrophages accumulate during plaque formation while M2 macrophages are present in the adventitia of normal arteries and linked to plaque regression. We found that *CXADR* mRNA was correlated strongly with *CD11b* (*r* = 0.6832; *P* < .001) and *CD11c* (*r* = 0.7579; *P* < .001), markers associated with M1 macrophages and dendritic cells (Table 1). Strong correlation between CXADR and CD11b (*r* = 0.87; *P* < .001) or CD11c (*r* = 0.87; *P* < .001) was also detected at protein levels (Supplementary Figure 2*A* and 2*B*). In comparison, CXADR was correlated moderately with CD163, a marker associated with M2 macrophages, at both mRNA (*r* = 0.5748; *P* < .001) and protein (*r* = 0.46; *P* < .01) levels (Table 1 and Supplementary Figure 2*C*).

**Table 1.** Correlation of Messenger RNA Levels Between Coxsackievirus and Adenovirus Receptor and Cell-Specific Markers in Human Carotid Plaques

Cell-Specific Markers	Gene	Pearson *r* Value	*P* Value
Macrophages/DCs			
CD68	*CD68*	0.6671	<.001
CD11b	*ITGAM*	0.6832	<.001
CD11c	*ITGAX*	0.7579	<.001
CD163	*CD163*	0.5748	<.001
CD36	*CD36*	0.7017	<.001
Lymphocytes			
CD27	*CD27*	0.1978	.03
CD28	*CD28*	0.4387	<.001
CD3 delta	*CD3D*	0.3317	<.001
CD4	*CD4*	0.3623	<.001
CD8A	*CD8A*	0.1279	.15
CD69	*CD69*	0.3168	<.001
ITGAE (CD103)	*ITGAE*	0.4701	<.001
Endothelial cells			
VE-cadherin	*CDH5*	0.2109	.02
von Willebrand factor	*VWF*	0.1793	.04
P-selectin	*SELP*	0.06395	.48
E-selectin	*SELE*	0.3188	<.001
Smooth muscle cells			
Myosin heavy chain 11	*MYH11*	−0.4886	<.001
Smoothelin	*SMTN*	−0.4408	<.001
Alpha smooth muscle actin	*ACTA2*	−0.3734	<.001
Myocardin	*MYOCD*	−0.4053	<.001
Transgelin	*TAGLN*	−0.4726	<.001

Significant cutoff level: P < .05.

Abbreviations: DCs, dendritic cells; ITGAE, integrin alpha E; VE, vascular-endothelial.

In recent reports, it has been revealed that macrophages developing into foam cells by oxidized low-density lipoprotein treatment display a dendritic cell–like phenotype including the expression of CD11c [27]. Based on our data, we reasoned that CXADR expression in CD68-positive cells could be related to foam cell formation. In support of this, we found that CXADR was correlated strongly with CD36, a scavenger receptor mediating cellular uptake of oxidized low-density lipoprotein and playing a key role in foam cell formation, at both mRNA (*r* = 0.7017; *P* < .001) and protein (*r* = 0.86; *P* < .001) levels (Figure 3C). In contrast, *CXADR* was correlated poorly with markers for lymphocytes and endothelial cells and negatively with markers for smooth muscle cells (Table 1).

### Induction of CXADR During Monocyte-to-Macrophage Differentiation

The results prompted us to further investigate whether CXADR was linked to macrophage differentiation and polarization. We compared the expression of CXADR with CCAAT-enhancer binding protein (C/EBP) and NF-κB transcription factors, which are known to play roles in macrophage differentiation and polarization. We found that *CXADR* was correlated strongly with *C/EBPβ* (*r* = 0.6391; *P* < .001) (Supplementary Table 1), a driver of monocyte-macrophage differentiation, which recently was implicated as a promoter of atherosclerosis [28–30]. Moderate correlation was observed between CXADR and C/EBP-β at protein levels (*r* = 0.45; *P* < .02) (Supplementary Figure 2*D*). In comparison, CXADR was correlated moderately with C/EBP-α (*r* = 0.5606; *P* < .001) and weakly with NF-κB1 (*r* = 0.3975; *P* < .001) and NF-κB2 (*r* = 0.3735; *P* < .001) (Supplementary Table 1).

**Figure 4.**
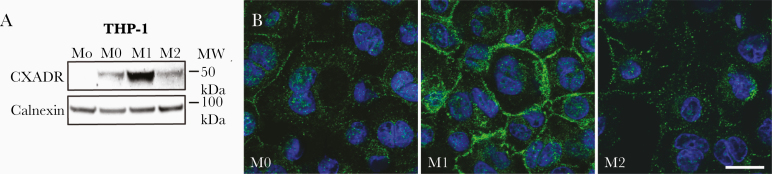
Induction of coxsackievirus and adenovirus receptor (CXADR) during monocyte-to-macrophage differentiation. *A,* Western blot analysis showing CXADR protein levels in THP-1 cells cultured as monocytes (Mo), differentiated into macrophages (M0), and further polarized into M1 or M2 macrophages. Abbreviation: MW, molecular weight. *B,* Immunofluorescence staining for CXADR (*green*) in THP-1 cells differentiated into M0, M1, or M2 macrophages. Cell nuclei were counterstained with DAPI (*blue*). Images were obtained with a Zeiss LSM700 confocal microscope and processed with NIS-Elements software (version 4.0). (https://www.microscope.healthcare.nikon.com/products/software/nis-elements). (Scale bar represents 10 μm.)

To study whether CXADR is regulated during macrophage differentiation we used a standard protocol, in which human THP-1 monocytes (Mo) were differentiated into macrophages (M0) by treatment with 50 ng/mL phorbol-12-myristate-13-acetate (PMA) for 3 days. Western blot analysis showed that while CXADR was not detected in undifferentiated Mo cells, it was induced in M0 cells after PMA treatment (Figure 4A and Supplementary Figure 3). To determine whether CXADR expression was affected by macrophage polarization we exposed PMA-treated M0 macrophages to combinations of either lipopolysaccharide (100 ng/mL) plus interferon γ (20 ng/mL) or interleukin 4 plus interleukin 13 (both 20 ng/mL), to polarize them into M1 or M2 macrophages, respectively. Compared with M0 macrophages, CXADR protein levels were increased in M1- but not M2-polarized macrophages (Figure 4A). These findings were supported by immunofluorescence staining showing increased cell surface staining of CXADR in M1 versus M0 and M2 THP-1 macrophages (Figure 4B). The results showed that CXADR was induced during monocyte-macrophage differentiation and further enhanced during M1 polarization.

### Association Between CXADR Expression in Plaques and Receptors for Other Viruses Linked to Atherosclerosis

**Figure 5.**
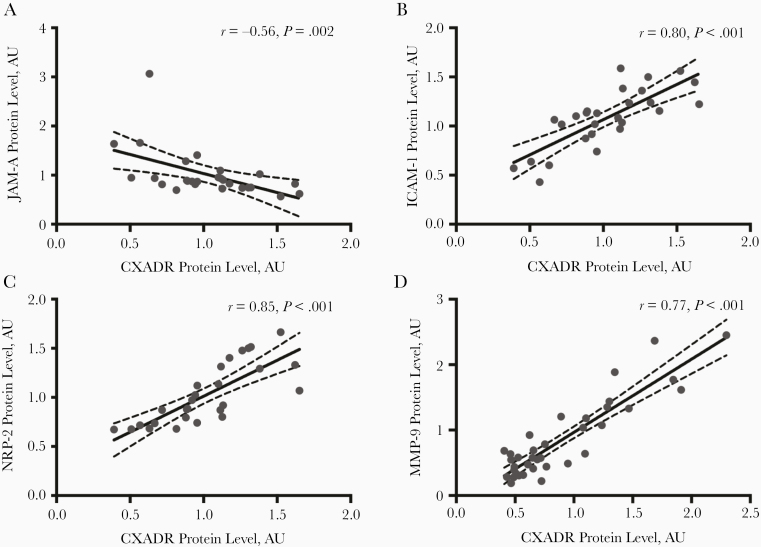
Proteomic analysis of the levels of coxsackievirus and adenovirus receptor (CXADR) and other virus receptors in human carotid plaques. *A–D,* Pearson correlation between CXADR and junction adhesion molecule A (JAM-A) (*A*), inflammatory cell adhesion molecule 1 (ICAM-1) (*B*), neuropilin 2 (NRP2) (*C*), and matrix metalloproteinase 9 (MMP-9) (*D*) protein levels in human plaques. Abbreviation: AU, arbitrary units.

Next, we studied to what extent CXADR expression was linked to other cell adhesion molecules and inflammatory markers in human plaques. Junction adhesion molecule A (*F11R*), which also is up-regulated in atherosclerotic plaques [31], was the only CTX member that was correlated strongly with *CXADR* (*r* = 0.7272; *P* < .001) (Supplementary Table 2). However, the correlation was negative at protein levels (*r* = −0.56; *P* = .002) (Figure 5A), suggesting that CXADR and junction adhesion molecule A are expressed in different cell types or differently regulated posttranslationally. Other junction components were correlated poorly with CXADR (Supplementary Table 2). In contrast, inflammatory cell adhesion molecule 1 (ICAM-1) was correlated strongly with CXADR, at both mRNA (*r* = 0.7016; *P* < .001) and protein (*r* = 0.80; *P* < .001) levels (Figure 5B). ICAM-1 is enriched in macrophages in advanced plaques [32] and contributes to plaque formation in mouse models of atherosclerosis [33]. Similar to CXADR, ICAM-1 is a virus receptor, for rhinoviruses [34], which cause respiratory infections but have been linked to myocardial infarction [35].

Further analysis showed that only a few inflammatory markers were correlated strongly with *CXADR* in plaques (Table 2). One of these was chemokine receptor 5 (CCR5), a receptor for HIV [36] (*r* = 0.6766; *P* < .001). CCR5 promotes initiation and progression of atherosclerosis by mediating macrophage recruitment [36]. The other was matrix metalloproteinase 9 (*r* = 0.77; *P* < .001) (Figure 5D), which has been linked to plaque rupture, and influenza virus infection [37]. Some inflammatory markers showed moderate correlation with CXADR including interleukin 1 β, CCL2, CCR2, transforming growth factor B1, tissue inhibitor of metalloproteinase 1, sulfatase 2, and caspase 3 (Table 2). Moreover, CXADR was correlated strongly with neuropilin 2 (*r* = 0.85; *P* < .001) (Figure 5C), a recently identified receptor for cytomegalovirus.

**Table 2.** Correlation of Messenger RNA Levels Between Coxsackievirus and Adenovirus Receptor and Inflammatory Markers in Human Carotid Plaques

Inflammatory Markers	Gene	Pearson *r* Value	*P* Value
Cytokines			
TNF-α	*TNFA*	0.2122	.02
IFN-γ	*IFNG*	0.02472	.78
IL-1 β	*IL1B*	0.5241	<.001
IL-2	*IL2*	0.08896	.32
IL-6	*IL6*	0.2385	.007
IL-4	*IL4*	−0.1683	.06
IL-5	*IL5*	−0.0368	.68
IL-9	*IL9*	−0.1354	.13
IL-10	*IL10*	0.3465	<.001
Chemokines and receptors			
MCP-1	*CCL2*	0.465	<.001
RANTES	*CCL5*	0.3258	<.001
CCR2	*CCR2*	0.4518	<.001
CCR5	*CCR5*	0.6766	<.001
CCR7	*CCR7*	0.3976	<.001
Growth factors			
TGF-β1	*TGFB1*	0.5662	<.001
TGF- α	*TGFA*	0.3581	<.001
IGF-1	*IGF1*	0.3001	<.001
PDGF-A	*PDGFA*	−0.2335	.008
PDGF-B	*PDGFB*	0.3694	<.001
PDGF-C	*PDGFC*	−0.2293	.01
PDGF-D	*PDGFD*	−0.3218	<.001
Matrix/degradation			
MMP-2	*MMP2*	−0.09967	.27
MMP-9	*MMP9*	0.6777	<.001
TIMP-1	*TIMP1*	0.5481	<.001
Sulfatase 2	*SULF2*	0.479	<.001
Apoptosis			
Caspase 3	*CASP3*	0.4494	<.001
Caspase 7	*CASP7*	0.2185	.01
Caspase 9	*CASP9*	0.05095	.57
BCL-2	*BCL2*	−0.2384	.007
BMP-4	*BMP4*	−0.2571	.004

Significant cutoff level: P < .05.

Abbreviations: BCL, B-cell lymphoma; BMP, bone morphogenetic protein; IFN, interferon; IGF, insulinlike growth factor; IL-1 β (etc), interleukin 1β (etc); MCP, monocyte chemoattractant protein; MMP, matrix metalloproteinase; PDGF, platelet-derived growth factor; TGF, transforming growth factor; TIMP, tissue inhibitor of metalloproteinase; TNF, tumor necrosis factor.

## DISCUSSION

Based on the documented links between enterovirus and atherosclerosis we hypothesized that plaque formation in arteries might be associated with altered expression or localization of the enterovirus receptor CXADR. Indeed, we found that CXADR was up-regulated during plaque formation in arterial walls and identified macrophages as a novel cellular source of CXADR in the plaque microenvironment. Further studies revealed that CXADR is induced during monocyte-to-macrophage differentiation and particularly associated with M1 macrophages and foam cells. Intriguingly, CXADR expression in plaques was also correlated specifically with receptors for several other types of viruses linked to atherosclerosis.

To our knowledge, these are the first results showing CXADR expression in cells derived from the hematopoietic system, which is somewhat surprising considering that CXADR was identified >2 decades ago. However, it might be explained by the results showing that not all CD68-positive cells in plaques stained positive for CXADR, and that CD68-positive cells in the appendix actually were negative for CXADR. Thus, CXADR expression does not seem to be a general feature of CD68-positive cells but may rather be specific to M1-polarized macrophages and foam cells accumulating in the inflammatory, lipid-rich milieu in atherosclerotic plaques. In comparison, CD68 expression is not constant in all macrophages and is induced during macrophage-foam cell conversion [38], which could explain why CXADR expression in plaques was correlated significantly with CD68 mRNA, but not with protein levels. The results warrant further studies to elucidate the role of CXADR in foam cells.

CXADR was also correlated with C/EBP-β, a key factor for monocyte-to-macrophage differentiation [28, 39], which is in line with our recent identification of CXADR as a C/EBP-β target gene [40] and suggests that C/EBP-β might be a driver of CXADR expression in plaque-associated macrophages. However, other mechanisms may also be important for CXADR regulation during macrophage polarization and foam cell formation. On this notion, it has recently been shown that subpopulations of foam cells in atherosclerotic plaques originate from intimal smooth muscle cells transdifferentiating into macrophagelike cells [41]. Our results showed a significant inverse relationship between CXADR and smooth muscle cell markers, and it will be interesting to study whether CXADR expression is regulated in smooth muscle cells transdifferentiating into macrophagelike cells.

An interpretation of our results is that M1 macrophages and foam cells in plaques are susceptible to infection with CVB and other enteroviruses using CXADR as a receptor. This might be counterintuitive because enteroviruses have a natural tropism for epithelial cells in the respiratory and gastrointestinal tracts. However, CVB and other respiratory viruses can disseminate via the systemic circulation, and, given that vascular permeability is increased in atherosclerotic vessels [42], it is tempting to speculate that circulating virus particles may extravasate in such areas and thereby get access to CXADR-positive macrophages. Such infections would likely trigger inflammatory responses that could lead to phagocytosis and cell death—factors associated with the development of a necrotic core, plaque disruption and myocardial infarction.

Interestingly, CXADR was correlated specifically with receptors for several other types of viruses linked to atherosclerosis. CCR5, a coreceptor for HIV, was recently found to play a role in the initiation and progression of atherosclerosis [36]. Similar to CXADR, CCR5 is also a target gene of C/EBP-β [43] and induced during monocyte-to-macrophage differentiation [44]. Coinfection with coxsackievirus and HIV has been reported in cardiomyopathy [45]. The scavenger receptor and foam cell marker CD36 is a coreceptor for hepatitis C virus, another viral pathogen associated with atherosclerosis [46]. CD36 is also induced during monocyte-to-macrophage differentiation [47] and is regulated by C/EBP transcription factors [48].

ICAM-1 is the main receptor for rhinoviruses [34], which, similar to other viruses causing respiratory infections, are significantly linked to myocardial infarction [35]. ICAM-1 is overexpressed in macrophages in atherosclerotic lesions [32] and contributes to plaque formation in *Apoe*^*−/−*^ mice [33]. CD11b is a surface receptor for viral double-stranded RNA, which is released from virus-infected cells and potently activates macrophages [49]. Matrix metalloproteinase 9 is induced during rhinovirus infection and is linked to advanced atherosclerosis [23]. Neuropilin 2, which was recently identified as a high-affinity receptor for cytomegalovirus, is also induced during monocyte-to-macrophage differentiation [50]. Future studies will show to what extent macrophages in atherosclerotic plaques are susceptible to infections and coinfections with viruses associated with atherosclerosis.

In summary, the results show that the enterovirus receptor CXADR is up-regulated in macrophages in human and murine atherosclerotic plaques. CXADR expression in plaques was specifically associated with M1 macrophages and foam cells, and a cluster of receptors for various viruses linked to atherosclerosis and myocardial infarction. Although many questions remain, the results provide new insight into how enterovirus may infect cells in atherosclerotic plaques.

## Supplementary Data

Supplementary materials are available at *The Journal of Infectious Diseases online*. Consisting of data provided by the authors to benefit the reader, the posted materials are not copyedited and are the sole responsibility of the authors, so questions or comments should be addressed to the corresponding author.

jiaa418_suppl_Supplementary_Material

## Notes

***Financial support.*** This work was supported by the Swedish Cancer Society (J. F.), the Swedish Research Council (grants 2017–02630 to J. F. and 2017-01070 to U. H.), the Diabetic Foundation, the Swedish Heart and Lung Foundation, the Strategic Cardiovascular Programs of Karolinska Institutet, the Stockholm County Council (U. H.), the Swedish Society for Medical Research (fellowship to L. M.), the Swedish Heart and Lung Foundation (fellowship to L. M.), and the Swen and Ebba Hagberg, Tore Nilsson, Magnus Bergvall, and Karolinska Institutet foundations (research funding to L. M.).

***Potential conflicts of interest.*** All authors: No reported conflicts. All authors have submitted the ICMJE Form for Disclosure of Potential Conflicts of Interest. Conflicts that the editors consider relevant to the content of the manuscript have been disclosed.
