# Supplementary material for: Induction of the Coxsackievirus and Adenovirus Receptor in Macrophages During the Formation of Atherosclerotic Plaques
Source: J Infect Dis. 2020 Jul 10;222(12):2041–51. doi: 10.1093/infdis/jiaa418 (PMC7661765; doi:10.1093/infdis/jiaa418)
Supplement: jiaa418_suppl_Supplementary_Material [file jiaa418_suppl_supplementary_material.docx]

# The Coxsackie- and Adenovirus Receptor is Induced in Macrophages During the Formation of Atherosclerotic Plaques

**Azadeh Nilchian^1,2^, Estelle Plant^2,4^, Malgorzata M. Parniewska^1^, Ana Santiago^2^, Aránzazu Rossignoli^3^, Josefin Skogsberg^3,5^, Ulf Hedin^4^, Ljubica Matic^4,7^ and Jonas Fuxe^1, 2, 7,^***

^1^Department of Laboratory Medicine, Division of Pathology, Karolinska Institutet, Stockholm

^2^Department of Microbiology, Tumor and Cell biology, Karolinska Institutet, Stockholm

^3^Department of Medical Biochemistry and Biophysics, Karolinska Institutet, Stockholm

^4^Department of Molecular Medicine and Surgery, Karolinska Institutet, Stockholm

^5^Current address: Université libre de Bruxelles

^6^Current address: Boehringer-Ingelheim AB, Stockholm

^7^Equal contribution

*****Corresponding author:

Jonas Fuxe, PhD

Department of Microbiology, Tumor and Cell biology (MTC)

Karolinska Institutet, SE-17177 Stockholm, Sweden

Phone: +46707980065; Email: [jonas.fuxe@ki.se](mailto:jonas.fuxe@ki.se)


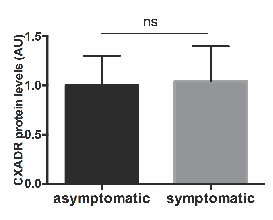


**Figure S1.** Proteomic analysis of CXADR in carotid plaques from symptomatic versus asymptomatic patients.

**Table S1. Correlation between *CXADR* and transcription factors in human plaques.**

| **Transcription factors** |  |  |  |  |
| --- | --- | --- | --- | --- |
| CEBPB | *CEBPB* | 0,6391 | <0,0001 | **** |
| CEBPA | *CEBPA* | 0,5606 | <0,0001 | **** |
| NFKB1 | *NFKB1* | 0,3975 | <0,0001 | **** |
| NFKB2 | *NFKB2* | 0,3735 | <0,0001 | **** |

**Figure S2. Proteomic data from human plaques.** (**A-D**) Pearson correlation analysis showing correlation between CXADR and JAM-A (**A**), ICAM-1 (**B**), NRP2 (**C**), and C/EBPβ (**D**) protein levels.

**Table S2. Correlation between *CXADR* and other CAMs in human plaques.**

|  | **Gene** | **Pearson r** | **P value** | **Significance** |
| --- | --- | --- | --- | --- |
| ***CTX-like CAMs*** |  |  |  |  |
| CLMP | *CLMP* | -0,0914 | 0,3068 | ns |
| ESAM | *ESAM* | -0,4231 | < 0,0001 | **** |
| JAM-A | *F11R* | 0,7272 | < 0.0001 | **** |
| JAM-B | *JAM2* | -0,1663 | 0,0616 | ns |
| JAM-C | *JAM3* | -0,5227 | < 0.0001 | **** |
| JAM-L | *AMICA1* | 0,5571 | < 0.0001 | **** |
| ***Junction-associated CAMs*** |  |  |  |  |
| Claudin-1 | *CLDN1* | -0,3151 | 0,0003 | *** |
| Claudin-3 | *CLDN3* | -0,03604 | 0,6875 | ns |
| Claudin-5 | *CDLN5* | 0,1084 | 0,2249 | ns |
| E-cadherin | *CDH1* | 0,2763 | 0,0017 | ** |
| N-cadherin | *CDH2* | -0,5222 | <0,0001 | **** |
| Occludin | *OCLN* | -0,2418 | 0,0062 | ** |


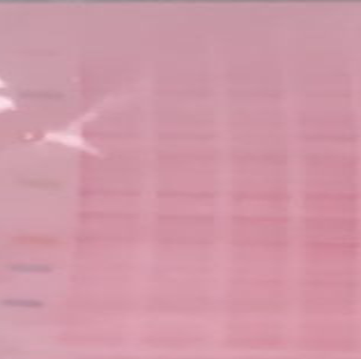


Marker

**A**

**B**

Mo

M0

M1

M2

M0

M0

M1

M1

Mo

M2


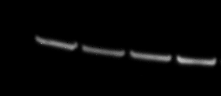


20s

Calnexin

100 kDa

Calnexin blot

70 kDa

50 kDa


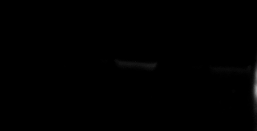


Mo

M2

CXADR blot

CXADR

30s


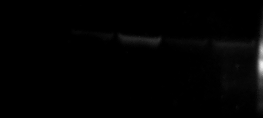


60s


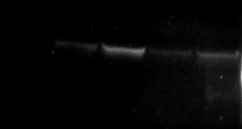


90s

**Figure S3.** Original gel blots from western blot results presented in Fig. 3A. (**A**) Ponceau staining of full-length membrane. Dashed lines indicate where membrane was cut for blotting with antibodies against CXADR (46 kDa) and calnexin (97 kDa). (**B**) Different exposure times for Calnexin (20s) and CXADR (30s, 60s and 90s) blots. The Spectra Multicolor Broad Range Protein Ladder from Thermofisher (cat. No. 2662) was used for the blotting experiments (Marker).
